# Effects of re-warm-up protocols on the physical performance of soccer players: A systematic review with meta-analysis

**DOI:** 10.5114/biolsport.2023.116013

**Published:** 2022-06-01

**Authors:** Francisco Tomás González Fernández, Hugo Sarmento, Álvaro Infantes-Paniagua, Rodrigo Ramirez-Campillo, Sixto González-Víllora, Filipe Manuel Clemente

**Affiliations:** 1 Department of Physical Activity and Sport Sciences, Pontifical University of Comillas. CESAG, 07013 Palma, Spain; 2 SER Research Group, Pontifical University of Comillas. CESAG, 07013 Palma, Spain; 3 University of Coimbra, Research Unit for Sport and Physical Activity. Faculty of Sport Sciences and Physical Education, Coimbra, Portugal; 4 Faculty of Education of Albacete, Department of Physical Education, Arts Education, and Music, University of Castilla-La Mancha. 02071 Albacete, Spain; 5 Human Performance Laboratory. Department of Physical Activity Sciences. Universidad de Los Lagos. Santiago, Chile; 6 Centro de Investigación en Fisiología del Ejercicio. Facultad de Ciencias. Universidad Mayor. Santiago, Chile; 7 Exercise and Rehabilitation Sciences Laboratory. School of Physical Therapy. Faculty of Rehabilitation Sciences. Universidad Andres Bello. Santiago, Chile; 8 Escola Superior Desporto e Lazer, Instituto Politécnico de Viana do Castelo, Rua Escola Industrial e Comercial de Nun’Álvares, 4900-347 Viana do Castelo, Portugal; 9 Instituto de Telecomunicações, Delegação da Covilhã, Lisboa 1049-001, Portugal

**Keywords:** Soccer, Football, Performance, Sports training, Warm-up, Neuromuscular, Vertical heigh jump, Linear sprinting, Half-time

## Abstract

This systematic review aimed to (1) identify and summarize studies that have examined the effects of re-warm-up (RWU) protocols on the physical performance of soccer players (vertical jump height and sprint time) and (2) establish a meta-comparison between performing a re-warm-up and not performing one regarding the outcomes of the aforementioned outcomes. A systematic review of EBSCO, PubMed, SciELO, SPORTDiscus, and Web of Science databases was performed on 12 January, 2021, according to the Preferred Reporting Items for Systematic Reviews and Meta-Analyses (PRISMA) guidelines. From the 892 studies initially identified, four studies were reviewed, and three of these were included in the present meta-analysis. Compared to a control condition, there was a moderate effect of RWU on vertical jump height (ES = 0.66; p = 0.001; I^2^ = 0.0%). However, compared to a control condition, there was a trivial effect of RWU on linear sprint time (ES = 0.19; p = 0.440; I^2^ = 38.4%). The nature of RWU enhances the performance of players with an emphasis on actions requiring vertical jumps. Therefore, the results provide essential information that soccer coaching staff can use to improve the performance of their teams. The limited number of studies available for the meta-analysis may have magnified the impact of heterogeneity on linear sprint time findings. More high-quality studies, with homogeneous study designs, may help to clarify the potential benefits of RWU for linear sprint time.

## INTRODUCTION

In soccer, re-warm-up (RWU) may increase players’ readiness to perform actions at different intensity levels [1] at the start of the second half of a match, thereby enhancing their athletic performance [2]. Indeed, RWU strategies have demonstrated that soccer players’ acute explosive performance improved during the first minutes of the second half of the match [3–5].

A review of the literature reveals that the physical performance of soccer players decreases during the second half of the match [6]. Common examples of this evidence can be found in studies showing poorer performances for different variables, such as the number of high-intensity actions and the total distance covered in the second half in comparison with the first half [7, 8]. Traditionally, these effects have been linked to changes in muscle and core temperatures [9] induced by passive half-time practice [10]. Nevertheless, improvements in physical performance in the second half have also been associated with RWU based on post-activation potentiation and supplementation with carbohydrate and caffeine [11]. Therefore, the direction of these physiological changes depends on the active behaviours performed during the 15 minutes of rest between halves [12].

Player’s capability of keep high values of physical strength plays an important role in the performance of soccer players in terms of their maximal strength, jumping ability, and sprint performance [7, 13] and decrease over the course of a match. In this sense, it is widely known that efficient neuronal activation generates more applicated weight reflected in vertical jump height, and improves the change-of-direction and linear sprinting performance in elite soccer players [14]. On the one hand, the higher level of fatigue generated during the first half and the team’s passive behaviour negatively impact players’ sprint and jump performance at the beginning of the second half. On the other hand, the implementation of RWU is positively related to an improvement in the performance at the beginning of the second half [15].

Even though there is evidence supporting the influence of acute effects of RWU on the physical performance of soccer players, described in a systematic review [4–6], little is known about its effect on certain physical variables of the game, such as vertical jump height, and sprint time. In fact, the most recent systematic review in RWU (as far as we know), centred the analysis in a myriad of performance analyses without a quantitative synthesis in the form of a meta-analysis [5]. Therefore, the aim of this systematic review was twofold: (i) to identify and summarize studies that have examined the effects of RWU protocols on the physical performance of soccer players (vertical jump height and sprint time) and (ii) to establish a meta-comparison between performing and not performing an RWU protocol regarding vertical jump height and sprint time.

## MATERIALS AND METHODS

### Design and protocol

The systematic review strategy was conducted according to Preferred Reporting Items for Systematic Reviews and Meta-analyses (PRISMA) guidelines [16]. The protocol was registered with the International Platform of Registered Systematic Review and Meta-Analysis Protocols with the number 202110055 and DOI: 10.37766/inplasy2021.1.0055.

### Eligibility criteria

The inclusion and exclusion criteria can be found in Table 1 according to the PICOS approach [17].

**TABLE 1:** Inclusion and exclusion criteria

	Inclusion criteria	Exclusion criteria
Population	Soccer players of any age or sex with no injury or illness, with normal vision, no partial/chronic injury or illness, and no history of neuropsychological impairment.	Population other than soccer players or members of the soccer player population with special conditions (e.g., injury, treatment, illness, diseases).

Intervention	RWU protocols (always performed after an initial warm-up) conducted under one of the two following possible conditions: after the warm-up and before the match. between the halves of the match.	Warm-up protocols.

Comparator	Passive control conditions.	Intervention conditions other than passive conditions.

Outcome	Vertical jump height, and sprint time	Physiological or physical conditions not related to the included outcomes.

Study design	Counterbalanced cross-over design (randomized and non-randomized can be included since none of them reveal significant differences in control conditions).	Study designs that do not allow within-subjects comparisons for the two conditions (control and RWU).

Additional criteria	Only original and full-text studies written in English.	Studies written in any language other than English. Articles other than original research (e.g., reviews, letters to editors, trial registrations, proposals for protocols, editorials, book chapters, and conference abstracts).

### Information sources and search

Five electronic databases (EBSCO, PubMed, SciELO, SPORTDiscus, and Web of Science) were explored for relevant publications prior to 29 January 2022. The Web of Science database search was performed restricting the search to the area of “sport sciences”. Keywords and synonyms were entered in various combinations in the title, abstract or keywords: (soccer OR football) AND (“re-warm-up” OR “post-warm-up” OR “warm-up” OR “pre-activity” OR “post-activation potentiation” OR “stretch*”) AND (“jump*” OR “sprint*” OR “change-of-direction” OR “agility”).

The searches, the removal of duplicates, screening of titles and abstracts, and analysis of the full texts in an independent way were performed by two authors (FTGF and AIP). The inter-rater agreement was measured through Cohen’s kappa [17], which was found to be good (kappa = 0.70). Any discrepancy in the selection process was solved by consensus with a third author (HS).

### Data extraction

An ad-hoc Microsoft Excel sheet (Microsoft Corporation, Redmond, WA, USA) was used to assess inclusion requirements in accordance with the Cochrane Consumers and Communication Review Group’s data extraction template [18]. The Excel sheet was used to assess inclusion requirements and subsequently tested for all selected studies. The process was independently conducted by two authors (HS and FTGF). Any disagreement regarding study eligibility was resolved in a discussion. Full text articles excluded, with reasons, were recorded. All the records were stored in the sheet.

### Data items

The outcomes chosen for this systematic review and meta-analysis included vertical jump height (VJH) and sprint time (ST). The VJH (measured in cm) was usually assessed during a countermovement jump (CMJ) with or without arm swing. The linear ST (measured in seconds) at different distances was also collected, without including values of partial times. Additionally, the following data items were extracted: (i) type of study design, number of participants (n), age group (youth, adults or both), sex (men, women or both), competitive level, moment of the season; (ii) characteristics of the RWU protocol (duration and intensity); (iii) moment of the match (after warm-up and between halves); (iv) characteristics of the experimental approach to the problem, procedures and settings of each study.

### Methodological assessment

The included studies were assessed according to their methodological quality with the Risk of Bias 2 (RoB 2) tool for RCTs [19]. We conducted a risk of bias assessment using the RoB-2 tool for RCTs [20] and the ROBINS-I for nonrandomized interventions [21]. These tools allow one to assess the risk of bias (i.e., “low risk,” “some concerns,” or “high risk”) of several dimensions, which vary according to the study design (namely, bias arising from the randomization process, bias due to deviations from intended interventions, bias due to missing outcome data, bias in measurement of the outcome, and bias in selection of the reported result). Altogether an overall level of risk of bias per study was computed. Risk of bias assessments were based on the published articles. Two of the authors (FTGF and AIP) independently screened and assessed the included articles. The inter-rater agreement was very good (Cohen’s kappa = 0.99). Discrepancies were solved by consensus between the two authors.

### Summary measures, synthesis of results, and publication bias

Although meta-analyses can be done with as few as two studies [22], considering the fact that reduced sample sizes are common in the sports science literature [23], meta-analysis was only conducted in the present case when three or more studies were available for the same outcome. Effect sizes (ES; Hedge’s *g*) for each outcome (i.e., linear sprint; vertical jump) in the experimental and control groups were calculated using pre-intervention and post-intervention mean and standard deviation (SD) for each outcome. Data were standardized using post-intervention SD values. The random-effects model was used to account for differences between studies that might impact the intervention effect [24–25]. The ES values are presented with 95% confidence intervals (95% CIs). Calculated ES were interpreted using the following scale: < 0.2, trivial; 0.2–0.6, small; > 0.6–1.2, moderate; > 1.2–2.0, large; > 2.0_4.0, very large; > 4.0, extremely large [26]. In studies including more than one intervention group, the sample size in the active control group was proportionately divided to facilitate comparisons across multiple groups [27] The impact of heterogeneity in the findings was assessed using the *I*^2^ statistic, with values of < 25%, 25–75%, and > 75% representing low, moderate, and high levels of heterogeneity, respectively. The risk of reporting bias was explored using Egger’s test [28], with *p <* 0.05 implying bias. To adjust for risk of reporting bias, a sensitivity analysis was conducted using the trim and fill method [29], with *L*_0_ as the default estimator for the number of missing studies [30]. All analyses were carried out using the Comprehensive Meta-Analysis software (Version 2.0; Biostat, Englewood, NJ, USA). Statistical significance was set at p < 0.05.

## RESULTS

### Study identification and selection

**FIG. 1:**
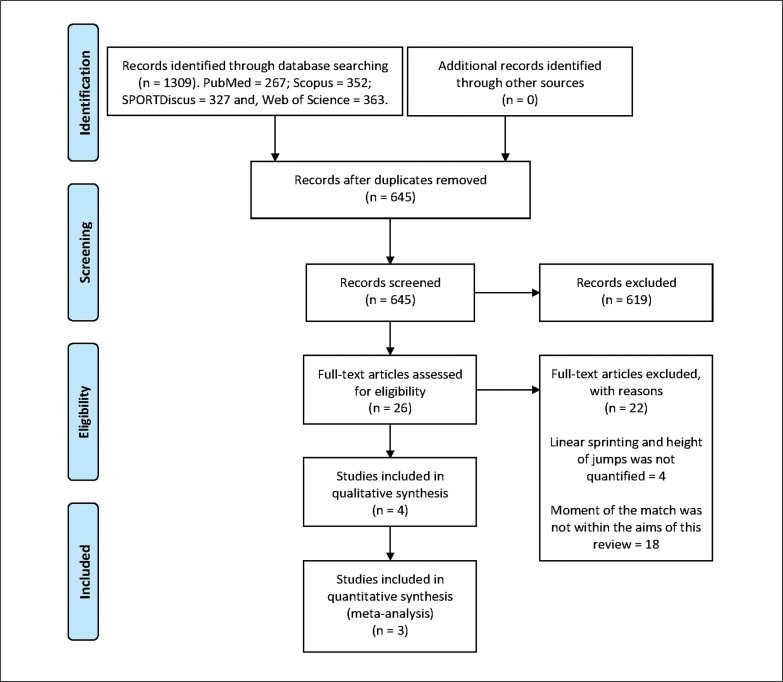
PRISMA flow diagram highlighting the selection process for studies.

The search of databases identified a total of 1309 titles (PubMed = 267; Scopus = 352; SPORTDiscus = 327; Web of Science = 363). These studies were then exported to reference manager software (EndNote X9, Clarivate Analytics, Philadelphia, PA, USA). Duplicates (664 references) were subsequently removed either automatically or manually. The remaining 645 articles were screened for their relevance based on the information contained in titles and abstracts and considering the selection and exclusion criteria (see Table 1). This resulted in the removal of a further 619 studies. Following the screening procedure, 26 articles were selected for in depth reading and analysis. After reading full texts, four studies were selected (Figure 1).

### Methodological quality

Table 2 shows the overall methodological quality assessment of the cross-sectional studies. Two out of three studies were classified as presenting high risk of bias studies, based on their overall RoB 2 quality scale [9, 31], and the other one was considered as having some concerns [32], while another study was classified as showing serious risk of bias in ROBINS-I [15]. Some issues were found regarding the quality of the information reported on the randomization process, the reporting of possible deviations from the intended interventions, the treatment of missing outcome data, and the selection of the reported result.

**TABLE 2:** Methodological assessment of the included studies.

Study	Outcome	D1a	D1b	D2	D3	D4	D5	DS	Overall
Edholm et al. [15]*	All	S	L	M	L	NI	M	M	S
Fashioni, Langley & Page [31]	All	SC	–	SC	H	L	SC	L	H
Lovell et al. [32]	All	SC	–	SC	L	L	SC	L	SC
Mohr et al. [9]	All	SC	–	SC	H	L	SC	–	H

*Note.* D1a: Randomization process; D1b: Timing of identification or recruitment of participants in a cluster-randomized trial; D2: Deviations from the intended interventions; D3: Missing outcome data; D4: Measurement of the outcome; D5: Selection of the reported result; DS: Period and carryover effects. C: Critical; H: High risk; L: Low risk; M: Moderate; NI: No information; S: Serious; SC: Some concerns.

* Assessment from ROBINS-I: D1a: Bias due to confounding; D1b: Bias in selection of participants for the study; D2: Bias in classification of interventions; D3: Bias due to deviations from intended interventions; D4: Bias due to missing data; D5: Bias in measurement of outcomes; DS: Bias in selection of the reported result.

### Characteristics of individual studies

The characteristics of the included studies and details of the RWU protocols can be found in Table 3.

**TABLE 3:** Characteristics of selected studies

Study	Age (years) Mean ± SD	Sample size *(n)*	Level / Sex	RWU characteristics	Design	Outcomes	
						Vertical jump height	Sprint time
**Mohr et al. [9]**	27.0. ± 1.5	8♂ CG 8♂ EG	Male Soccer players competing in the Danish 4^th^ Division	Re Warm Up1: 15′ rest (control) Re Warm Up2: 7′ rest + 7′ jog + exercises at 135 beat/ min	Randomized (participants) Parallel groups	-	Re Warm Up2 > Re Warm Up 1 *d* = −0.66 – 0.67% Test (Repeated sprint test. 30 m sprint)

**Lovell et al. [32]**	20 ± 1	10♂	Semi-professional male soccer players	Re Warm Up1: rest (control) Re Warm Up2: 9′ rest + 5′ Intermittent Agility Exercise Re Warm Up3: 9′ rest+5′ Whole Body vibration at 40 Hz	Counterbalanced Crossover design.	Re Warm Up2 > Re Warm Up 1 Test (CMJ)	Re Warm Up2 > Re Warm Up1 Test (10 m sprint)

**Edholm et al. [15]**	Range 18–33	22♂	Twenty-two male professional soccer players	Re Warm Up1: traditional half-time break Re Warm Up2: 7′ rest + 7′: jog and calisthenics	Crossover design.	Re Warm Up2 > Re Warm Up1 *d* = 0.29 3.02% Test (CMJ)	Re Warm Up2 > Re Warm Up1 *d* = −0.72 – 2.02% Test (10 m sprint)

**Fashioni, Langley & Page [31]**	23 ± 4	10♂	Male Amateur Soccer Players	Control Trial: 15-min Half Time Passive Re Warm Up Trial: 12 min + 3 min Re Warm Up.	Cross-over design.	Re Warm Up improvement in squat (d = 0.6; CON: 26.96 ± 5.00 CM; RWU: 30.17 ± 5.13) II) CMJ = (d = 07; CON: 28.15 ± 4.72 CM; RWU: 31.53 ± 5.43) Test (CMJ and AJ)	Re Warm Up improvement in 20 m sprint times. (d = 0.6; CON: 3.42 ± 0.20 S; RWU: 3.32 ± 0.12). Test (5 m, 10 m and 20 m sprint)

Three out of the four studies followed a cross-over design [15, 31, 32], and the other one was a randomized parallel-groups study [9]. Regarding the participants, all studies were performed with male soccer players [9, 15, 31, 32]. The level of participants was amateur in two studies [9, 32], whereas one study included semi-professional players [15], and the other one involved professional players [31]. In addition, sample sizes varied from 10 participants [15, 32] to 22 participants [31]. The parallel-group study included 16 participants (eight in each group) [9]. Lastly, concerning the studies’ RWU protocols, all studies employed a traditional RWU during the 15-min resting period between match halves while approaching the active RWU (7′ rest + 7′ jog + exercises at 135 beats/min [9], 9′ rest + 5′ intermittent agility exercises [15], 7′ rest + 7′ jog and calisthenics [31] and 12′ + 3′ RWU [32]).

Sprint time was measured in all studies [9, 15, 31, 32], through repeated sprint test, 30 m [9], 10 m sprint [15, 32], and 5 m, 10 m and 20 m sprint [31]. Non-significant effects of RWU were found on 30 m sprint [9]. In fact, positive effects of RWU were found on 10 m sprint [15, 32]. Last, 20 m sprint improved after RWU, but non-significant effects were found on 5 m and 10 m [31].

Vertical jump height was assessed with CMJ [15, 31, 32] and squat jump (SJ) [31]. Significant positive effects of RWU protocols were found on CMJ [15, 31, 32] and SJ [31].

### Effects of re-warm-up on vertical jump height

**FIG. 2:**
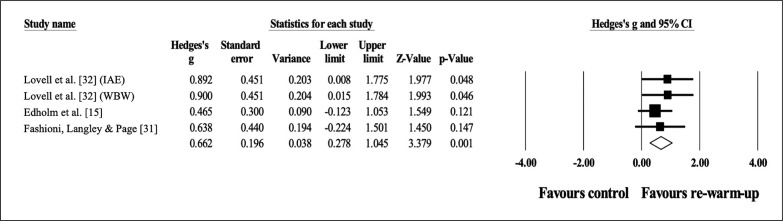
Forest plot of effects of re-warm-up on vertical jump height compared to a control condition.

Three studies provided data for vertical jump height (pooled n = 104). Compared to a control condition, there was a moderate effect of RWU on vertical jump height with no heterogeneity (ES = 0.66; 95% CI = 0.28 to 1.05; *p* = 0.001; I^2^ = 0.0%; Egger’s test *p* = 0.098; Figure 2). The weight used in each study in the analysis ranged from 18.8% to 42.5%.

### Effects of re-warm-up on sprint time

**FIG. 3:**
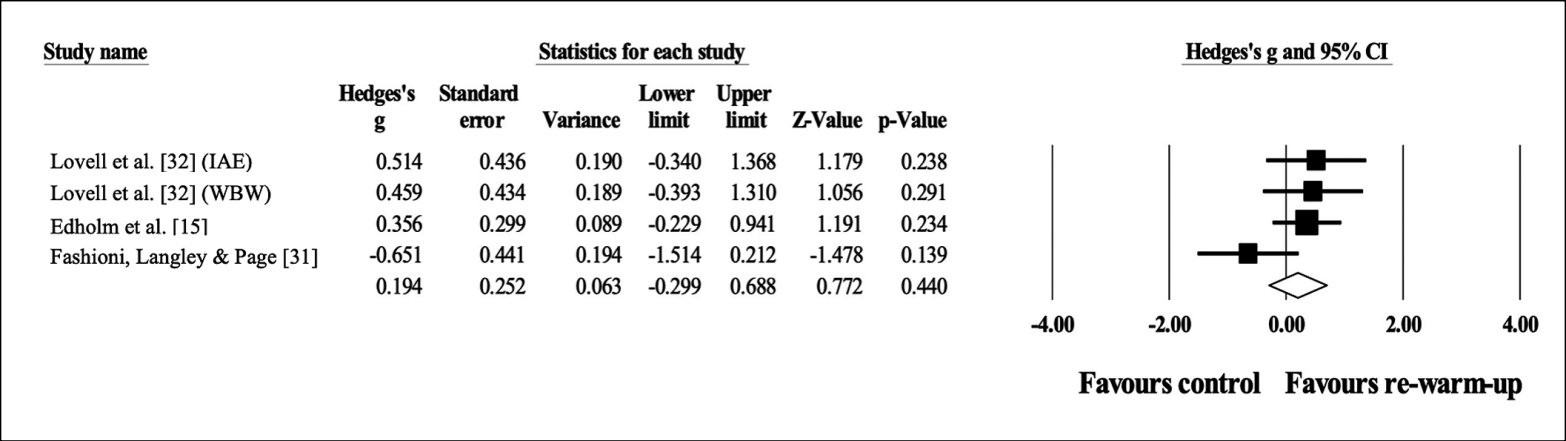
Forest plot of effects of re-warm-up on sprint time compared to a control condition.

Three studies provided data for linear sprint time (pooled n = 104). Compared to a control condition, there was a trivial effect of RWU on linear sprint time with moderate heterogeneity (ES = 0.19; 95% CI = -0.30 to 0.69; p = 0.440; I^2^ = 38.4%; Egger’s test p = 0.692; Figure 3). The relative weight of each study in the analysis ranged from 21.8% to 34.0%.

## DISCUSSION

The aim of this systematic review with meta-analysis was to determine the effects of RWU protocols on the vertical jump height and sprinting performance of soccer players. The meta-analysis revealed a significant and moderate beneficial effect of RWU on players’ vertical jump height, although no significant benefits were found in sprinting performance.

### Effects of RWU on vertical jump height

Traditionally, the countermovement jump (CMJ) has been explored in different ways (See Morin et al., 2019 for more information [33]), namely as a physical quality associated with lower-limb power [34] and as a readiness marker to detect the influence of fatigue on neuromuscular properties [35]. In the case of soccer, CMJ has also been confirmed as a determinant of other qualities such as sprinting or change of direction [36, 37]. Since CMJ is dependent on the recruitment of higher-order motor units, potentiation mechanisms, and/or muscle activation [38, 39], it can be expected that warming up plays an important role in immediate performance maximization.

The meta-analysis presented in this systematic review revealed moderate and significant beneficial effects of RWU protocols on CMJ. These changes were identified using the p-value and effect size. However, in a practical scenario, it would be necessary to analyse the raw data for the smallest worthwhile change. However, in this particular review, we did not have access to the raw data of included articles, which justifies the option for using the p-value and effect size to sustain the evidence of the beneficial effects of RWU on CMJ. Although the protocols of exposure to RWU were generally based on RAMP (raise, activate, mobilize, and potentiate), the effects were large enough to signify the importance of using RWU to preserve players’ jumping performance immediately before the match re-start. Although post-activation potentiation using heavy loads appears to represent a better neuromuscular stimulus for enhancing vertical jump [40], the protocols consisting of running, dynamic stretching, and straight sprinting [15, 31]; intermittent agility exercises [32]; or whole-body vibrations [32] had a favourable effect on the reduction the decremental effects of rest on vertical jump performance.

It is reasonable to hypothesize that elevated muscle temperatures improve the recruitment of motor units, possibly justifying the beneficial effects of RWU [15]. Additionally, considering that meaningful decreases in eccentric hamstring strength can be observed after inactivity during half-time [32], a re-activation before the second half of a match is also recommended to enhance vertical jump height, which requires the potentiation of muscles to increase players’ performance.

### Effects of RWU on sprint time

Sprinting is one of the most important high-intensity demands in soccer. Although it rarely occurs during matches [41], this action is crucial since it is related to decisive moments of matches that precede goal-scoring opportunities [42]. Usually, sprints last 2–4 s over distances shorter than 20 m [43]. Since sprinting performance depends on neuromuscular readiness and neural activation [44], the proper physiological conditions (e.g., induced by warming up) should be ensured [45]. Warming up is expected to immediately benefit sprinting performance, considering that it plays an important role in fully activating the working musculature and recruiting motoneurons to support the high magnitude of contractions [9, 46]. Considering that the half-time break presents a long period of rest/inactivity, it can induce a drop in muscle temperature (~1.5°C), thus negatively impacting sprinting performance [9].

The meta-analysis conducted in the current systematic review revealed a trivial and non-significant beneficial effect of RWU on sprinting performance. Among the included studies, Lovell et al. [32] and Edholm et al. [15] reported a beneficial and significant effect of RWU strategies in comparison to control conditions. Lovell et al. [32] stated that temperature was higher in the RWU group, possibly contributing to the players’ muscle readiness for sprinting. However, neuromuscular facilitation caused by reflex potentiation could be another physiological cause of this benefit of RWU [46]. In a different study, the effects of RWU on sprinting performance were positive immediately after exposure, as well as ten and 15 minutes after exposure. Again, additional factors related to fatigue during the match could have impacted the comparisons [43]. Therefore, the literature provides relevant information about the requirements for high level soccer players and specific details to ensure the stability and solidity of second-half performance.

Generally, the individual studies emphasize the benefits of RWU for mitigating the decline in sprinting performance induced by the half-time rest period. Thus, from a practical point of view, coaches can choose to use a combined approach such as jogging, skipping, dynamic stretching, and straight sprinting [15, 31], or implement intermittent agility exercises [32], or use whole-body vibrations [32], or even selected alternative strategies such as potentiation-post activation based on lifting moderate-to-high loads [47] or short high cycling efforts [48].

### Study limitations, future research, and practical implications

This systematic review has some limitations. One is the small number of included articles, which should be considered a limiting factor regarding the generalization of evidence. For example, the impact of heterogeneity (i.e., I^2^ = 0.0%) on the vertical jump height results was low. In contrast, a greater impact of heterogeneity (i.e., I^2^ = 38.4%) affected the linear sprint time findings. The limited number of studies available for meta-analysis may have magnified the impact of heterogeneity on linear sprint time findings. More high-quality studies, with homogeneous study designs, may help to clarify the potential benefits of RWU for linear sprint time. Another limitation is that only articles written in English were included; thus, some relevant literature may have been excluded. However, the results found are promising regarding the implementation of RWU strategies in soccer players. Eventually, short, moderate-to-high demanding activities should be ensured to maximize the increase in muscle temperature and neuromuscular activation in the shortest time possible, thus saving some time for the recovery during half-time [5].

Future research should consider determining the most adequate and practicable RWU strategies for soccer players, namely by comparing post-activation performance enhancement strategies, RAMP, whole-body vibration, or cycling. Duration of exposure and an analysis of this parameter’s impact on the first minutes of the match should also be integrated into future reports. Determining the intensity and identifying thresholds should also be considered, namely for individualizing these strategies. As a consequence, more research is needed on the effects of RWU, as the dose-response effects on individual soccer players need to be better understood in order to achieve the best possible performance.

As example, the time of RWU is critical. As suggested in a study conducted in twelve amateur players comparing the effects of a 10- and 20-minute regular warm-up [49], a detrimental effect of the longer period on the muscular power output was found, followed by excessive thermal discomfort and fatigue. Similar evidence was found in a study comparing 8-, 15- and 25-minute warm-up protocols, in which only the condition of 8 minutes ensured significant improvements in the acceleration ability of soccer players [50]. Thus, considering these examples, time-efficient and optimal RWU strategies should be compared in crossover study designs. However, the saving of time should not compromise the effectiveness of RWU for improving some protective factors such as stabilization, balance, or extremity symmetries. As an example, a study using the regular warm-up of FIFA11+ revealed the potential of this condition for improving unilateral jumping and dynamic balance and reducing lower limb extremity symmetries in female soccer players [51].

For now, it is possible to argue only that RWU strategies do not cause negative effects in comparison to control conditions and that they have a favourable effect on vertical jump height. For soccer coaching staff, more evidence is needed on the cost-benefit ratio of implementing an RWU in their match routines, eventually considering the extent of benefits to the first high-intensity demands occurring in the re-start of matches.

A practical application of this review is the improvement of substitution decision-making in high-performance matches [52, 53]. In fact, if soccer coaching staff have information about the evidence of RWU, they will be able to make much more effective player changes according to the specific tactics with which they intend to change the dynamics of a match (e.g., which player to send out when their team is trailing by one goal and there are 25 minutes left in the match). In this case, it would be appropriate to put players with high jumping power on the pitch, as this could be beneficial for finishing or heading shots.

In turn, this review provides key information when it comes to signing players and building a balanced squad, as the factors that have been shown to be positive can be taken into account to select the best players for a top-level team.

## CONCLUSIONS

The most relevant contribution of this work is that there is a significant and moderate beneficial effect of RWU on vertical jump height, but not on sprinting performance, in soccer players. This information is crucial for soccer coaching staff to improve the performance of their teams. Given the small number of publications found, this research line can be described as emerging, as more studies are needed to consolidate the scientific evidence found.
